# Comparison of laser and power bleaching techniques in tooth color change

**DOI:** 10.4317/jced.53435

**Published:** 2017-04-01

**Authors:** Reza Fekrazad, Shervin Alimazandarani, Katayoun AM Kalhori, Hadi Assadian, Seyed-Mahdi Mirmohammadi

**Affiliations:** 1 DDS, Msc, Associate professor. Department of Periodontology, Dental Faculty - Laser research center in medical Sciences, AJA University of Medical Sciences, Tehran, Iran. International Network for Photo Medicine and Photo Dynamic Therapy (INPMPDT), Universal Scientific Education and Research Network (USERN), Tehran, Iran; 2 DDS, Private Practice, Tehran, Iran; 3 MSc, Iranian Medical Laser Association, Tehran, Iran; 4 DDS, MSc, Assistant Professor, Department of Endodontics, Shahed University, and Tehran, Iran; 5 DDS, Medical Laser Research Center, ACECR, Tehran, Iran

## Abstract

**Background:**

Laser-assisted bleaching uses laser beam to accelerate release of free radicals within the bleaching gel to decrease time of whitening procedure. The aim of this study was to compare the efficacy of power bleaching using Opalescence Xtra Boost® and laser bleaching technique using LaserSmile gel and diode laser as an activator in their tooth whitening capacity.

**Material and Methods:**

Student t test showed that the laser bleaching group significantly outperformed the power bleaching group in changing ∆E (*p*=0.977).

**Results:**

Similarly, while comparing the groups in changing ∆L, the laser bleaching group indicated significantly superior results (*p*=0.953). Statistical data from student t test while comparing the groups in changing the parameter of yellowness indicated that samples in laser bleaching group underwent a more significant reduction than power-bleached samples (*p*=0.85). Correspondingly, changes in whiteness were statistically tested through student t test, showing that laser bleaching technique increased whiteness of the samples significantly more than those treated by power bleaching (*p*=0.965). The digital color evaluation data was in accordance with spectrophotometry and showed that laser bleaching outperformed power bleaching technique. Both techniques were able to increase whiteness and decrease yellowness ratio of the samples. ΔE decrease for laser bleaching and power bleaching groups were 3.05 and 1.67, respectively. Tooth color change in laser bleaching group was 1.88 times more than that of power bleaching group (*p*<0.001).

**Conclusions:**

It could be concluded that under the conditions of this study, both laser-assisted and power bleaching techniques were capable of altering tooth color change, but laser bleaching was deemed a more efficient technique in this regard.

** Key words:**Laser, power bleaching, tooth color introduction.

## Introduction

Lightening tooth color can be successfully accomplished by a wide variety of bleaching methods, including in-office (professionally administered), at-home (professionally dispensed) and over-the-counter (self-administered) techniques. Home bleaching techniques require more procedural time and cannot be well controlled due to their mode of administration. The main advantages of in-office bleaching technique includes dentist control, avoidance of tissue exposure, reduced treatment time and enhanced patient satisfaction due to immediate results (Joiner 2006). Power bleaching has been performed using varying hydrogen peroxide activation techniques since 1910 (1). Serious concerns have been stated concerning the safety of conventional hydrogen peroxide- containing bleaching products, including enamel surface alterations including minute depressions, porosities and slight erosive defects following scanning electron microscopic evaluations (2,3). Considering the fact that pH of different brands of bleaching gels vary greatly (4), it is stated that in a laboratory setting hydrogen peroxide solution (pH=6.4) is able to remove mineral contents of enamel, although the critical pH for enamel demineralization has been considered to be between 5.2 and 5.8 (5).

Laser-assisted bleaching uses laser beam to accelerate release of free radicals within the bleaching gel to decrease time of whitening procedure. Lasing is also capable of minimizing post-bleaching hypersensitivity, loss of enamel microhardness and gingival irritation due to lack of hydrogen peroxide use. Decreased chair time can also be anticipated following laser use. Several diode laser systems with wavelengths ranging from 790 to 980nm have been used for laser-assisted bleaching (6-8). It has been shown that laser bleaching with the bleaching gel in place can help control increased intra-pulpal temperature (1,8-10).

Efficacy of power bleaching compared with laser bleaching in tooth color change has been studied by different authors with controversial results. It was shown that there was no considerable difference in whitening capacity between light-activated and non-activated bleaching systems (11,12). On the other hand, it has been shown that bleaching gel activation using different light sources such as halogen (400-500nm), infrared (2000-4000nm) and plasma lamp (400-550nm) could lead to a considerable success in total whitening compared with different laser systems i.e. argon ion laser(488nm), diode laser (830nm) and CO2 laser (10600nm) (11). Luk, Tam *et al.*, Gurgan *et al.* showed that diode laser-activated bleaching gels significantly outperformed power bleaching, using spectrophotometry (13).

It has been claimed that light can improve the effectiveness of some bleaching agents, while some others are not influenced (14,15). However, many authors have indicated that light energy does not have any clinical influence on tooth whitening (12).

Tooth whitening systems have been under a considerable attention for their efficacy and safety so that some concerns have been raised concerning tooth sensitivity as well as soft-tissue irritations (9,16-19). Controlled clinical trials focusing on the efficacy and safety of light-activated bleaching agents are limited (14). The aim of this study was to compare the efficacy of power bleaching using Opalescence Xtra Boost® and laser bleaching technique using LaserSmile gel and diode laser as an activator in their tooth whitening capacity.

## Material and Methods

In this experimental *in vitro* study 20 anterior teeth including freshly extracted maxillary central and mandibular lateral incisors were selected. The teeth were visualized under 10X magnification to rule out carious lesions, enamel cracks or surface irregularities. The buccal surface of all teeth were decontaminated by pumice and ProphyPaste( Dentsply International Inc, Milford, DE) . All samples were disinfected by immersion into 0.2% thymol solution for 48 hours and stored in normal saline.

-Sample Preparation 

The teeth were mounted into rose wax and randomly divided into two experimental groups (n=10 each).

In the first group (i.e. LB), bleaching was performed using LaserSmile gel (Biolase, Technology, Inc, San Clemente, Calif.) containing 35% hydrogen peroxide. The gel was activated by diode laser (λ= 810 nm) in 7- and 4-minute intervals, according to the manufacturer’s recommendation.

In the second group (i.e. PB), bleaching was performed using Opalescent Xtra Boost gel (Ultradent Products, South Jordan, UT) containing 38% hydrogen peroxide. The gel was chemically activated and applied in two separate 15- and 20-minute periods. The procedure was followed exactly according to the manufacturer’s guidelines. The samples in both groups were subsequently stored in normal saline. Non-etched protocol was contemplated for treatment and the samples were not polished following treatment.

-Color Evaluation

Evaluation of the color change was performed through two different methods:

a. Spectrophotometry

All samples were evaluated before and after treatment in Iranian Institution of Color. Color evaluation data prior to treatment was recorded as control for the data obtained after treatment. The buccal surface of each sample was fitted in front of a 1m X 2.5m rectangular window. The wall of the evaluation room was gray in color which could eliminate interference of penetrating beams. Inside the evaluator chamber was thoroughly white-colored to cause a homogenous emission without any beam qualification. The chamber was absolutely dark but after starting the machine, a single white beam was sent out to the tooth on its buccal surface and the resultant reflection was evaluated. Beam emission and sample maintenance was done automatically without any operator interference and the experiment was performed reproducibly. The apparatus was completely calibrated according to the universal standards. Data were recorded and analyzed by Optiview Lite (GretagMacbeth Instruments Corporation, USA).

Within the Optiview Lite software system some parameters were taken into consideration:

L*: which indicated brightness of the sample. Brighter samples (but not necessarily whiter ones) show increased L* values.

a*: which indicated redness of the sample and had no impact in our evaluation. This parameter should be in a more limited range (less than zero) according to the whiteness of the tooth surface.

b*: positive values indicated yellowness and negative ones blueness of the sample. Values greater than zero (positive values) indicate yellowness and decreased values show decreased yellowness. Values less than zero (negative values) show blueness which have nothing to do with tooth surfaces. Regarding the yellow hue of dentin, b* is commonly positive in a healthy tooth. However, ∆b might be positive and/or negative indicating increased or decreased yellowness, respectively.

**Figure 1:**
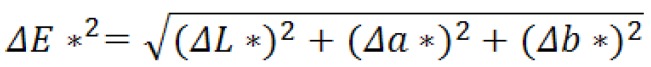
Equation.

ΔE*: indicated total color change. Values equal to or less than 1 showed that no color change occurred. Values more than one show more pronounced color change. ΔE* is a parameter related to the changes in the values of L*, b* and a* according to the following equation, (Fig. 1).

In addition to the aforementioned parameters, yellowness and whiteness of the samples were also taken into consideration.

b. Digital color evaluation

Both before and after treatment digital photographs were taken from each sample at 12.00 in sunny days in a semi-dark room with a 5.0 megapixel Sony CyberShot camera (Sony CyberShot, Tokyo, Japan) at a fixed object-lens distance (22cm). Digital photographs were transferred to computer and color evaluation was performed by Adobe Photoshop 6.0 for Windows. On the buccal surface of each sample 6 different points were evaluated and compared before and after the operation. Average values of the aforementioned six points were compared. Three colorimetric parameters were evaluated in this method as follows:

R domain; ranging from dark green (0) to light red (255)

G domain; ranging from dark purple (0) to light yellow (255)

B domain; ranging from dark yellow (0) to light blue (255)

## Results

 The colorimetric evaluation by Optiview Lite software was performed in both laser bleaching and power bleaching groups before and after treatment on the parameters of yellowness, whiteness, ∆L and ∆E ( Table 1).

**Table 1:**
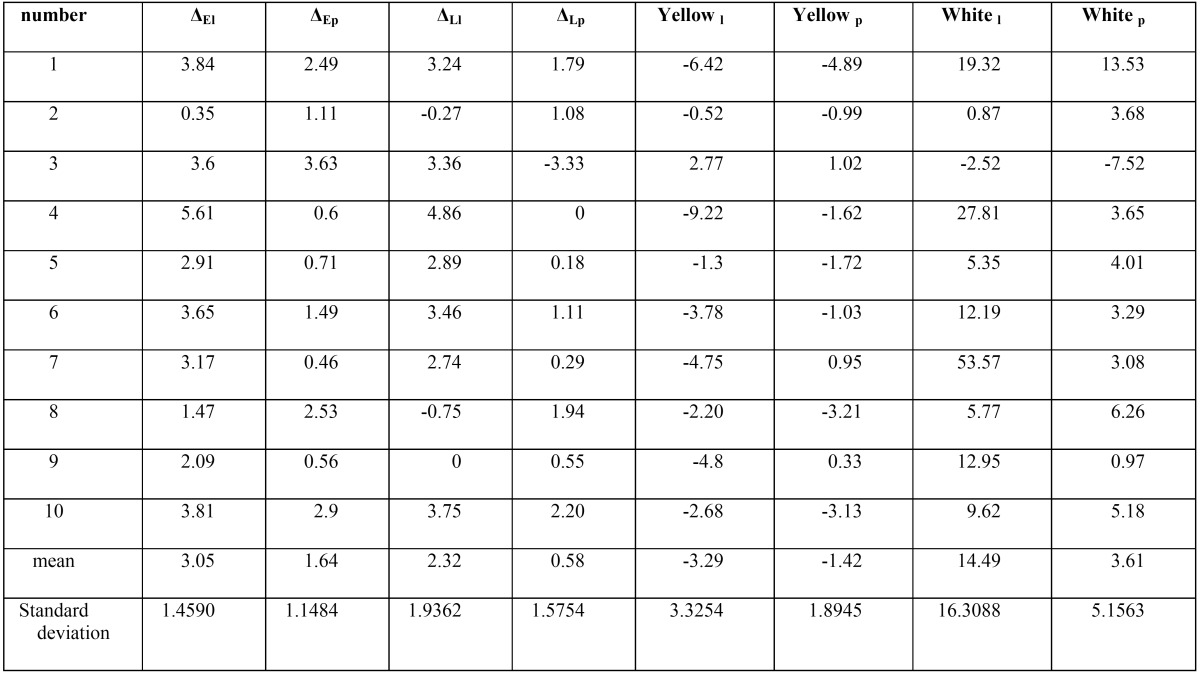
The colorimetric evaluation by Optiview Lite software was performed in both laser bleaching and power bleaching groups before and after treatment on the parameters of yellowness, whiteness, ∆L and ∆E.

Kolmonogorov-Smirnov statistical test revealed that the samples had a normal distribution at 95% confidence level (α=0.05). Student t test showed that the laser bleaching group significantly outperformed the power bleaching group in changing ∆E (*p*=0.977). Similarly, while comparing the groups in changing ∆L, the laser bleaching group indicated significantly superior results (*p*=0.953). Statistical data from student t test while comparing the groups in changing the parameter of yellowness indicated that samples in laser bleaching group underwent a more significant reduction than power-bleached samples (*p*=0.85). Correspondingly, changes in whiteness were statistically tested through student t test, showing that laser bleaching technique increased whiteness of the samples significantly more than those treated by power bleaching (*p*=0.965).

The digital color evaluation data was in accordance with spectrophotometry and showed that laser bleaching outperformed power bleaching technique.

Both techniques were able to increase whiteness and decrease yellowness ratio of the samples. ΔE decrease for laser bleaching and power bleaching groups were 3.05 and 1.67, respectively. Tooth color change in laser bleaching group was 1.88 times more than that of power bleaching group (*p*<0.001).

## Discussion

The aim of this study was to compare the efficacy of power- and laser bleaching techniques on tooth surface color change. Therefore extracted teeth were randomly categorized to be treated by laser or power beaching techniques and subsequently compared for their changes in colorimetric parameters.

Employing laser bleaching can reduce the operation time, thereby increasing patient comfort and cooperation. Treatment time in laser bleaching is 15 to 20 minutes, whereas power bleaching lasts 45 to 60 minutes. Laser has also been considered as the most valuable energy source for in-office bleaching (6).

It has been indicated that laser bleaching using diode laser (810nm) is a safer remedy compared with power bleaching using 38% hydrogen peroxide gel.

The ability of a light source to heat hydrogen peroxide can theoretically be considered as an advantage since it can increase the rate of forming oxygen free radicals from the bleaching agent and improving the release of stain containing molecules (20,21).

Several methods have been proposed to evaluate tooth color changes following whitening procedures. Spectrophotometry has been considered as a standard method for color evaluation. It has some advantages including lack of operator interference, reproducibility, reliability in calibration and excluding the environmental light interference (22). This technique is universally standardized and software-dependent in data recording and analysis, therefore minimizing personal evaluation bias.

The digital color evaluation has the advantage of reproducibility, but cannot eliminate the operator interference and is not calibrated well. This technique cannot be easily standardized. In this study, camera settings were performed manually and the resultant pictures were compared using a computer software. This evaluation was performed in accordance with spectrophotometry and was not statistically evaluated. The results obtained from this technique was in agreement with those of spectrophotometry showing that laser bleaching was more efficient in color change than power bleaching.

Color change evaluation using 3D Vita shade guide and Easy Shade spectrophotometry was also taken into consideration in this study but due to some disadvantages such as environmental light interference, lack of calibration and reproducibility, it was not employed in this investigation.

In spectrophotometric color evaluation in this study, two factors are extremely important including:

a. Yellowness-C: that if decreased, can show increased whiteness and decreased yellowness, and the reverse is also true.

b. Whiteness-Ganz: that if increased, can show increased whiteness, and the reverse is also true.

Additionally other parameters such as ∆b, ∆L and ∆E were also evaluated. ΔE* can show the efficacy of the method. Yellowness-C and whiteness-Ganz can directly evaluate whiteness of the samples. Δb* and ΔL* are less important than yellowness-C and whiteness-Ganz.

Although spectrophotometry is considered an objective method in determining tooth color change, it also has some disadvantages as well. For instance, it is influenced by tooth translucency, contour and texture. In addition, repeatable tooth positioning in this technique can be achieved with difficulty. In the current study, mounting teeth in acrylic blocks and determining a fixed position of the samples and spectrophotometer window could overcome this problem (13,15).

Some authors have questioned the use laser in activation of tooth whitening gels due to increased hypersensitivity and a non-significant difference when bleaching agent was not laser-activated (23). This might be attributable to the laser used for activation of the bleaching gel. On the other hand, a clinical investigation indicated that use of diode laser resulted in less dental and gingival sensitivity compared with non-activated bleaching agents (13). It has been shown that laser-assisted bleaching is more effective than an LED-activated system in terms of changes in chroma and luminosity (7).

## Conclusions

It could be concluded that under the conditions of this study, both laser-assisted and power bleaching techniques were capable of altering tooth color change, but laser bleaching was deemed a more efficient technique in this regard.
